# Evaluating Physiological Dynamics via Synchrosqueezing: Prediction of Ventilator Weaning

**DOI:** 10.1109/TBME.2013.2288497

**Published:** 2013-11-04

**Authors:** Hau-Tieng Wu, Shu-Shua Hseu, Mauo-Ying Bien, Yu Ru Kou, Ingrid Daubechies

**Affiliations:** 1 * Department of MathematicsStanford University Stanford CA 94305 USA; 2 Department of AnesthesiologyTaipei Veterans General Hospital Taipei 112 Taiwan; 3 School of Respiratory TherapyTaipei Medical University Taipei 110 Taiwan; 4 Division of Pulmonary MedicineDepartment of Internal MedicineTaipei Medical University Hospital Taipei 110 Taiwan; 5 Division of Pulmonary MedicineDepartment of Internal MedicineWan Fang Hospital Taipei 116 Taiwan; 6 * Institutes of Physiology and Emergency and Critical Care MedicineNational Yang-Ming University Taipei 112 Taiwan; 7 Department of MathematicsDuke University Durham NC 27705 USA

**Keywords:** Heart rate variability (HRV), instantaneous frequency, physiological dynamics, respiratory rate variability (RRV), synchrosqueezing transform, ventilation weaning prediction

## Abstract

Oscillatory phenomena abound in many types of signals. Identifying the individual oscillatory components that constitute an observed biological signal leads to profound understanding about the biological system. The instantaneous frequency (IF), the amplitude modulation (AM), and their temporal variability are widely used to describe these oscillatory phenomena. In addition, the shape of the oscillatory pattern, repeated in time for an oscillatory component, is also an important characteristic that can be parametrized appropriately. These parameters can be viewed as phenomenological surrogates for the hidden dynamics of the biological system. To estimate jointly the IF, AM, and shape, this paper applies a novel and robust time-frequency analysis tool, referred to as the synchrosqueezing transform (SST). The usefulness of the model and SST are shown directly in predicting the clinical outcome of ventilator weaning. Compared with traditional respiration parameters, the breath-to-breath variability has been reported to be a better predictor of the outcome of the weaning procedure. So far, however, all these indices normally require at least $\hbox{20}$min of data acquisition to ensure predictive power. Moreover, the robustness of these indices to the inevitable noise is rarely discussed. We find that based on the proposed model, SST and only $\hbox{3}$ min of respiration data, the ROC area under curve of the prediction accuracy is $\hbox{0.76}$. The high predictive power that is achieved in the weaning problem, despite a shorter evaluation period, and the stability to noise suggest that other similar kinds of signal may likewise benefit from the proposed model and SST.

## I. Introduction

Biological signals can contain a wealth of information. In particular, to evaluate a person's physiological condition we can extract information from a variety of biological signals such as ECG, respiratory signals, blood pressure, and circadian rhythm [1]–[8]. In some cases, this information is easy to read and interpret, in others, it is less accessible, and more sophisticated approaches are needed to extract the information. Many of the measured signals are oscillatory, and one particular and common technique is to focus on the oscillatory features. The fundamental quantity describing the oscillation is its period, which is defined to be the time needed for an observer to observe a “complete and intact oscillation”; this can be expressed by the frequency, which qualitatively is the inverse of the period, that is, it gives the number of oscillations per unit time period. For example, the periods of the ECG signal, the respiratory signal, and the circadian rhythm are about 1 s, 5 s, and 24 h, corresponding to $\hbox{1}$ Hz, $\hbox{0.2}$ Hz, and $\hbox{11.6}$ mHz, respectively [2]–[5]. In recent years, growing evidence suggests that information extracted from biological signals with oscillatory features has diagnostic and prognostic value in various diseases [1] [2] [4] [5] [6] [9] [10].

Mathematically, frequency analysis is typically studied via the Fourier transform when the signal can be assumed to be stationary. However, it has been long observed that the stationarity assumption is too restrictive for physiological signals, and more information of the physiological system can be extracted if one allows time dependence in the frequency or period. For example, the variability of the time intervals between sequential heart beats, or heart rate variability (HRV) [2] [4], observed in ECG signals, and the variability of the time intervals between sequential breath intakes, or respiratory rate variability (RRV) [3] [10] [11] [12] [13] [14] [15] [16] are well known to be related to physiological dynamics. Accurate extraction of this type of time-varying information improves diagnostic accuracy and treatment quality [3] [4] [9] [10] and much effort has been put on this direction. In general, time-varying frequency is not measured directly, but is inferred from the behavior, in time, of the oscillation-to-oscillation intervals. A well-known example is the analysis of R-peak to R-peak intervals (RRI) to reveal HRV information [4] [9] [17]. Many techniques have been introduced, including spectral methods and nonlinear dynamical analysis, such as Poincare map, entropy analysis, fractal analysis, to analyze these oscillation-to-oscillation intervals [10]–[17].

These established analysis techniques have at least the following three limitations, however, in their use for the study of physiological signals. A first limitation is the (relatively) large number of oscillations that must be observed. For example, for analysis of respiratory signals, at least $\hbox{300}$ and $\hbox{100--1000}$ oscillations are needed for methods that use a Poincare map [10] [14] [15] or approximate entropy [12] [18], respectively. It is feasible to collect many points for the ECG signal, since this requires only a continuous recording for about $\hbox{5}$ min, a reasonable length for a bedside observation. For signals that function on larger time scales than the ECG signal, the story is different. For example, for respiratory signals, we normally need at least $\hbox{20}$ min or longer to collect the necessary amount of data, which is usually difficult in certain clinical settings such as patients in intensive care unit [10] [12] [14] [15] [16] or newborns [18]. The situation would be even worse if we wanted to analyze larger time-scale physiological signals such as circadian rhythm [5] [7].

**Fig. 1.**
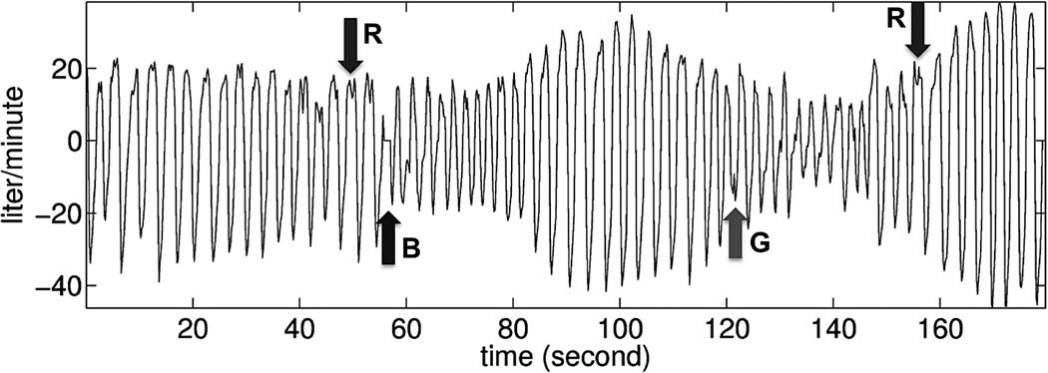
Typical recorded respiratory signal from an intensive care unit patient with support from a ventilator. The arrows with a mark R indicate times in the signal where it would be difficult to identify a basic oscillation if only peaks were taken into consideration; the arrow with a mark B indicates a brief machine recalibration; the arrow with a mark G indicates an invalid respiratory trigger. This patient succeeded in ventilator weaning.

The second limitation is that it is not always straightforward to determine the oscillation-to-oscillation time series from the given oscillatory signal. Recall that this determination depends on being able to isolate individual oscillations, which requires the identification of the complete repeating basic pattern and, possibly, landmarks within the pattern. Given a suitable definition of this repeating pattern, the oscillation-to-oscillation time series is determined by finding the landmarks for each oscillation. For example, for the ECG signal, the pattern is related to the electrophysiological activity of a normal heart beat and the landmark is defined to be the R peak, and the RRI-time series is based on the R-peak detection [4] [9] [17]. For other physiological signals, it is not always easy to define a basic “oscillation” or a “landmark,” even for healthy subjects. For example, although we can provide a definition for respiratory signals, in practice determining the landmarks is not easy, specifically when there is invalid or doubly triggered respiration (see Fig. 1). This difficulty can be mitigated to some extent by noise removal or noise reduction algorithms [17], but even then no reliable determination of the “true” landmarks can be guaranteed. Sometimes it is hard to even provide a universally accepted definition of a landmark, e.g., for the electroesophagographic signal.

The third limitation is the overreduction of the information inside the physiological signals by retaining only the oscillation-to-oscillation time series. For example, respiratory signal information is hidden in the time varying amplitude of the ECG signal [19], which is lost in the RRI time series. Indeed, the basic pattern of the oscillation of the ECG signal itself also varies according to time due to the cardiac axis rotation induced by the respiration and other effects.

To address these limitations, we propose in this paper a descriptive model featuring a more fine-grained description of the oscillatory physiological signal than given by the time intervals between sequential oscillations. The model is characterized by the wave-shape function, which is defined to replace the definition of an oscillation and the landmark, the instantaneous frequency, the variability of which is defined to be a proxy for the physiological dynamics, and the amplitude modulation, which is aimed to capture more physiological information. The companion algorithm, referred to as the synchrosqueezing transform (SST), is introduced to provide an accurate estimation of the instantaneous frequency and the amplitude modulation [20] [21].

We recently reported [14] [15] that small variabilities of respiratory parameters including rate and flow are associated with a high incidence of weaning failure in intensive care unit patients, and these variabilities may serve as reliable predictors for weaning patients from mechanical ventilation. As an testbed of the proposed model and algorithm, we analyze the respiratory signals collected from patients in one of our recent studies [15]. We show that the variation of the recorded respiratory signal, monitored for as brief a period as 3 min, together with the tidal volume information, can be used to define a weaning index (WIN) that predicts weaning process outcome with a success rate quantitatively as high as with the area under curve (AUC) of $\hbox{0.76}$ when analyzed by the receiver operation characteristic (ROC).

## II. Model and Methodology

### A. Model

In this section, we provide a phenomenological model describing general oscillatory physiological signals. A physiological system is closely linked with a variety of other physiological systems that interact in complex ways; it is well known that, for example, chemical set points and metabolic demand play a role in respiration patterns [3] [15]. Our treatment of these signals will be purely phenomenological; that is, the parameters and indices we will derive from observations of the physiological signal will be based solely on these signals themselves, and not on explicit, quantitative models of the underlying mechanisms. We will show by example in the next section that these parameters and indices in the model contain information that can provide insight into the functioning of the underlying physiology.

The major characteristic pattern of an oscillatory physiological signal is that it is a (fairly) periodic phenomenon; we therefore model it (without noise) as

$$f(t) = A(t) \, s(2\pi \phi (t)) \eqno{\hbox{(1)}}$$

where $s(\cdot)$ is a continuously differentiable periodic function we call the wave shape function, $s(t+2\pi)=s(t)$ for all $t$; it is an oscillating function that satisfies some mild technical conditions [22] (Note that to make the discussion clear, we assume that the signal has just one component, unlike [22], where superpositions of several components were considered.) We call the derivative $\phi^{\prime }(\cdot)$ of the phase function $\phi (t)$ the instantaneous frequency of $f$; we require it to be positive, but it need not be constant; we allow it to vary in time, as long as the variations are slight from one period to the next, i.e., $\vert \phi^{\prime \prime }(t)\vert \le \epsilon \phi^{\prime }(t)$ for all $t$, where $\epsilon$ is some small, preassigned number. Likewise, the amplitude $A(t)$ should be positive, but is allowed to vary slightly as well, i.e., $\vert A^{\prime }(t)\vert \le \epsilon \phi^{\prime }(t)$. In summary, we have the following conditions for all $t\in {\BBR}$:

$$A(t)>0,\,\,\, \phi^{\prime }(t)>0,\,\,\, \vert A^{\prime }(t)\vert \le \epsilon \phi^{\prime }(t),\,\,\, \vert \phi^{\prime \prime }(t)\vert \le \epsilon \phi^{\prime }(t). \quad\eqno{\hbox{(2)}}$$

For the identifiability problem raised in this model and hence the terminologies instantaneous frequency and amplitude modulation, we refer the reader to [21] for the discussion.

In reality, the recorded physiological signal $g(t)$ is contaminated by noise or measurement error, and we model the deviated physiological signal as [21] [23]

$$g(t) = f(t)+\sigma (t)\Phi (t) \eqno{\hbox{(3)}}$$

where $\Phi (t)$ is a generalized stationary random process with finite variance and $\sigma (t)$ is a slowly varying smooth function which capture the heteroscedasticity of the error. Although the possible noise appearing in the medical signal is versatile, our model covers a large portion of it, for example, the time-dependent noise, the Poisson noise and even “slightly” nonstationary noise. Note that the commonly used Gaussian white noise model is when $\sigma (t)=\hbox{1}$ and $\Phi (t)$ is the derivative of the Brownian motion.

### B. Methodology

Given the model (1) for the oscillatory physiological signal $f(t)$, we want to capture the time-varying quantities of the signal, including the instantaneous frequency $\phi^{\prime }(t)$ and the amplitude modulation $A(t)$, when the signal is contaminated by noise as the model (3). It is well known that the continuous wavelet transform (CWT) and short time Fourier transform (STFT) provide profound information about these time-varying quantities, in particular the instantaneous frequency, but accurate extraction via these methods remains an issue, even after many years of research. Reallocation is a technique widely employed in order to get accurate estimates of the instantaneous frequency [24]–[26]. In general, these methods reallocate the wavelet coefficients or STFT coefficients according to some “regrouping” rule, making it possible to read the instantaneous frequency from the resulting time-frequency plane representation.

Synchrosqueezing transform (SST) is a recently introduced novel reallocation technique introduced in [27] in order to analyze speech signals; it was theoretically proved to enjoy several nice properties, useful in our analysis of [20]–[23]. Specifically, the instantaneous frequency $\phi^{\prime }(t)$ and the amplitude modulation $A(t)$ can be accurately estimated and the estimation does not depend on whether or not the wave-shape function is a cosine [22]; moreover, the SST is robust to several different types of noise, like the white or colored Gaussian noise [23] or almost stationary generalized random process [21]. Furthermore, the analysis result is adaptive to the data in the sense that the error is dependent only on the first three moments of the chosen mother wavelet and its derivative instead of the profile of the mother wavelet. We summarize the reallocation technique and SST in Appendix A and its numerical implementation in Appendix B.

For the sake of convenience, in what follows we shall use the acronyms SSTIF (for SynchroSqueezing Transform-derived Instantaneous Frequency) to refer to the SST-estimated instantaneous frequency, and SSTAM (SynchroSqueezing Transform-derived Amplitude Modulation) to refer to the SST-estimated amplitude modulation.

## III. Testbed: Ventilator Weaning Problem

Making a weaning decision for a patient on a ventilator is clinically an important issue. The RRV has proved to be helpful in predicting the outcome of weaning intubated patients from the ventilator [14]–[16]. Extended intubation has many negative side effects, such as an increased risk for infection [28] [29]; ideally physicians seek to extubate as soon as medically possible. Yet, weaning too early carries risk as well: reintubation leads to stress to patients or a higher mortality rate [28] [29]). It is thus important to decide accurately when patients can be weaned from the ventilator. To increase the weaning success, the present common practice is to conduct spontaneous breathing trials before a weaning attempt; the final weaning decision is based on the patient's performance during spontaneous breathing trials, characterized by parameters derived directly from the respiration signal, such as the rapid shallow breathing index (RSBI) [30], and subjective evaluation by the clinician. Unfortunately, weaning failure still occurs in a significant percentage of patients who are judged ready-to-wean [28] [29]. Recently, several RRV-based predictors have been proposed to increase the rate of success weaning in this context; these newer predictors are reported to have a higher accuracy than RSBI in predicting weaning success or failure [10] [14] [15] [16]. Since the oscillation-to-oscillation time series is the focus of RRV analysis, observation of the respiration signal usually takes at least 20 min to guarantee highly significant prediction accuracy [13]–[15]. In addition, these more accurate RRV-based predictors rely on accurate timing of breath intake “peaks”, and are thus likely subject to stability issues caused by the inevitable noise.

**Fig. 2.**
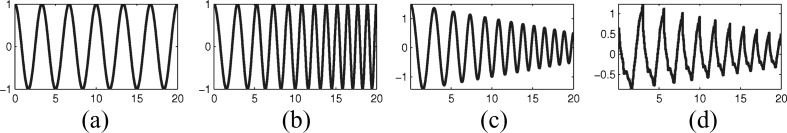
(Modeling the respiration signal) Panel (a) plots a cosine function, an overly simplified model of the respiration; moving to the right shows enrichment of this model by allowing nonconstant instantaneous frequency (b) amplitude modulation (c) and a more complex wave shape function (d). The (nonconstant) instantaneous frequency and the amplitude modulation function should capture how the oscillations vary in time, independent of whether the basic shape function is a simple trigonometric function or not.

**Fig. 3.**
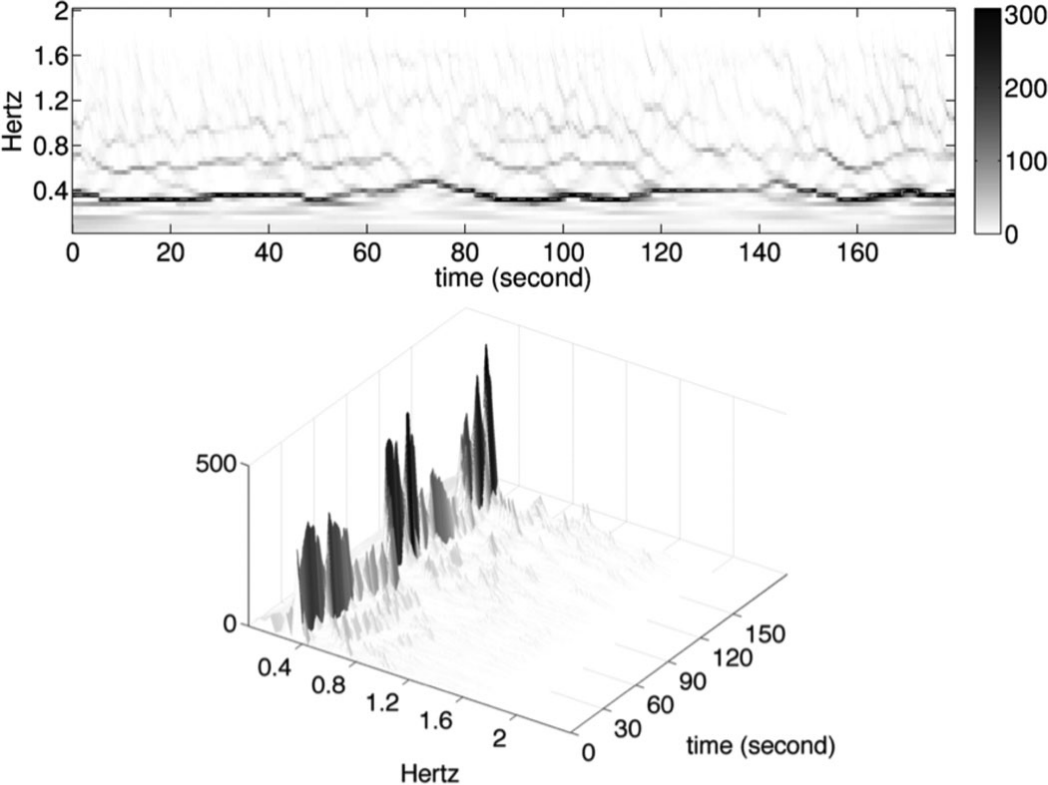
Top row: the result of the Synchrosqueezing transform (SST) of the signal in Fig. 1 with the SSTIF (dashed curve) superimposed; bottom row: the 3-D version of the SST result. The instantaneous frequency corresponds to the dominant curve in the SST, and the amplitude modulation corresponds to the intensity upon the dominant curve (visible in the 3-D graph). Indeed, the spacing of respiration cycle is reflected by SSTIF: closer spacing corresponds to higher SSTIF values, and wider spacing to lower SSTIF values; the darker curve corresponds to higher SSTAM values, and lighter curve to lower SSTAM values. In conclusion, through reading the SST figure, we can visually see how the frequency and amplitude modulation vary according to time.

To mitigate these limitations, we analyze the respiratory signal by the approach proposed in Section II. First, we model the respiratory signal $R(t)$ (without noise) as

$$R(t) = A(t) s(2\pi \phi (t)), \eqno{\hbox{(4)}}$$

where $s(\cdot), A(t)$, and $\phi (t)$ satisfy (2). Fig. 2 illustrates the role of the different constituents of f(t) modeling the respiratory signal. In reality, the recorded respiration signal ${\hbox{\tt {Resp}}}(t)$ is noisy, and we model it as

$$\hbox{\tt Resp}(t) = R(t)+\sigma (t)\Phi (t) \eqno{\hbox{(5)}}$$

where $\sigma (t)\Phi (t)$ satisfies the same conditions as that of (3). We apply the methodology described in Section II to directly analyze ${\hbox{\tt {Resp}}}(t)$ signals measured continuously during clinically feasible time intervals, far shorter than $\hbox{20}$ min. The SST result of the recorded respiratory signal demonstrated in Fig. 1 is shown in Fig. 3.

## A. Study Material

To validate the combination of the model and the SST algorithm in deriving the dynamics of a physiological signal with large oscillatory scale, we consider the following database collected in a recent study [15] for the purpose of studying the ventilator weaning problem.

All protocols in that study [15] were approved by the Institutional Review Board of Taipei Veterans General Hospital, Taipei, Taiwan, and written informed consent was obtained from patients. The study subjects were $\hbox{68}$ ready-for-weaning intubated patients collected in the intensive care unit of Taipei Veterans General Hospital, Taipei, Taiwan. In particular, all subjects are with RSBI ${\le}\hbox{110}$ breaths/min/L since we excluded patients not ready for weaning with RSBI ${>}\hbox{110}$ breaths/min/L before spontaneous breathing trial (SBT) due to the restriction from the Institutional Review Board. For each subject, we continuously recorded a $\hbox{30}$ min flow signal at the sampling rate $\hbox{100}$ Hz during SBT under the T-piece ventilator mode. The characteristics of these patients and the protocol to perform SBT are described in detail in [15]. These $\hbox{68}$ subjects are divided into weaning success ($n=\hbox{45}$) and failure ($n=\hbox{23}$) groups, based upon their extubation outcomes. Extubation was defined to be successful if patients did not need the ventilator again for at least $\hbox{48}$ h after extubation. Reinstitution of either noninvasive or invasive mechanical ventilation within $\hbox{48}$ h of extubation was considered an extubation failure. In [15], the data show no difference between the success and failure groups in the mean values of six clinically used weaning predictors measured before subject inclusion, and also in the mean values of three breathing pattern parameters measured after subject inclusion; in other words, the mean values of these nine clinically used weaning predictors could not allow us to discriminate success cases from failures.

## B. WIN Index

We now define the WIN index capturing the breathing pattern variability. Given the respiratory signal ${\hbox{\tt {Resp}}}(t)$, we apply SST to get the SSTIF and SSTAM. In the respiratory signal, the SSTAM can be understood as the instantaneous tidal volume. Then, we define the WIN index

$$\hbox{WIN}=\hbox{var}\left({\hbox{SSTAM}(t)\over \hbox{SSTIF}(t)} \right). \eqno{\hbox{(6)}}$$

**Fig. 4:**
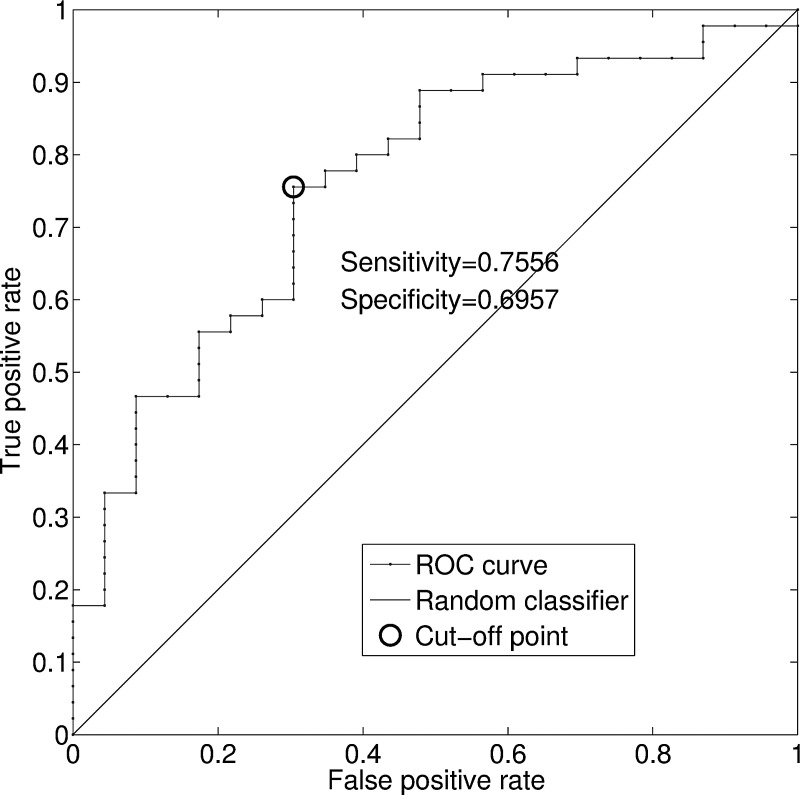
ROC curve of the WIN index in the dataset of $\hbox{68}$ patients. The cut-off point is marked in circle, where the WIN index value is $\hbox{88.8841}$, the sensitivity is $\hbox{0.7556}$, and the specificity is $\hbox{0.6957}$.

**Fig. 5.**
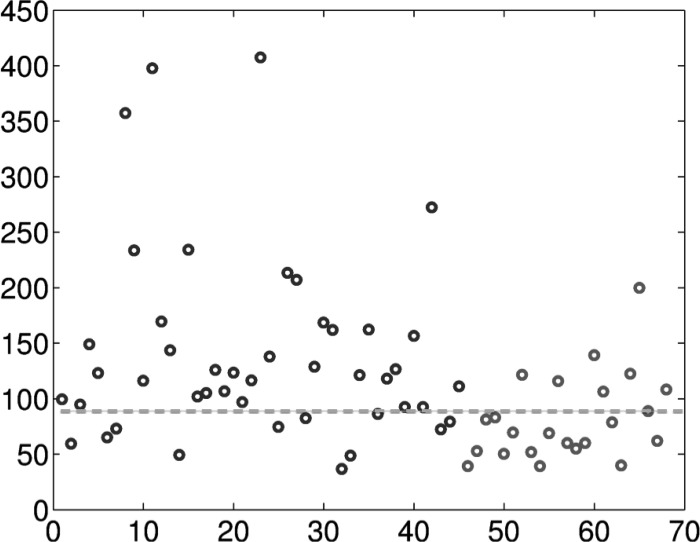
WIN indices of the $\hbox{68}$ patients. The black circles are the WIN indices for the patients who succeeded in the weaning procedure (numbered $\hbox{1}$ to $\hbox{45}$ here), while the gray circles are for the patients who failed in the weaning procedure (numbered $\hbox{46}$ to $\hbox{68}$ here). The gray dashed line is the cut-off value $\hbox{88.8841}$ determined by the ROC.

For all $\hbox{68}$ patients, the SST is applied to the first $\hbox{3}$ min respiratory flow signal during SBT under the T-piece mode, from which the WIN indices are evaluated. The WIN indices of each subject and the ROC curve are shown in Figs. 4 and 5. The AUC is $\hbox{0.76}$, and the $\hbox{95}\%$ confidence interval is $[\hbox{0.65}, \hbox{0.87}]$. The confidence interval is evaluated by $\hbox{1000}$ bootstrap replicas. Based on the analysis of the ROC curve, we obtained a cut-off value of the WIN index, which is $\hbox{88.8841}$ with a sensitivity of $\hbox{0.7556}$ and specificity of $\hbox{0.6957}$. The WIN indices of all $\hbox{68}$ patients are shown in Fig. 5. As shown, the cut-off value of WIN index could allow us to separate a majority of patients with success weaning procedure from those with failure weaning procedure. Accordingly, this cut-off value of WIN index might be helpful for the physicians to make decision which patients merit weaning from mechanical ventilation. Notice that our method requires only three consecutive minutes of respiratory signal observation. It is important to note that the occurrence, during the observation windows, of machine calibration or some breathing irregularities, such as coughs, invalid trigger, etc., which normally require special attention in the existing analyses, is not an impediment for our method. These properties of the SST for a patient in the success group are shown in Fig. 3 for demonstration.

## IV. Discussion

In this paper, we introduced a phenomenological model and the synchrosqueezing transform (SST) to alleviate the limitations of traditional methods for the analysis of oscillatory physiological signals. The usefulness of this combination is shown by applying it to the study of the ventilator weaning problem. Our results show that the SSTIF and SSTAM and their derived quantity WIN from the respiratory signal provide a suitable criterion for clinical use to predict weaning outcome, with an ROC-AUC of $\hbox{0.76}$.

At first sight, this result does not improve upon what has been reported for RRV-based predictions [14]–[16]. One should take into account, however, the following two points. First, the WIN requires only $\hbox{3}$ min of consecutively recorded data rather than the at least $\hbox{20}$ min typically required for the RRV-based indices; since a $\hbox{3}$ min-observation is entirely feasible in clinical practice, whereas a $\hbox{20}$ min-observation is much less so this is a crucial difference. Second, the definition of an oscillation and its landmark are not critical in the analysis. Note that even if the definition of an oscillation and its landmark are precisely given, the landmark detection is typically not robust under perturbations by noise, e.g., invalid breathing in the respiratory signal. Although the necessarily noisy signal can be “denoised,” existing noise removal or noise reduction algorithms cannot guarantee a reliable determination of the “true” landmarks. Even if “oscillation-based segmentation” could be carried out perfectly on a long enough recorded physiological signal so that enough oscillations are collected, it is not straightforward to obtain such an uninterrupted signal, in practice, especially when the period of each oscillation is large than, say, $\hbox{2}$ s. Different kinds of interruptions during the signal record, for example, machine calibration, coughs, suction, irritation, and so on, require the signal analyst to artificially cut-and-stitch together pieces of signal so that the oscillation-to-oscillation intervals can be defined, as needed for the traditional method, even though there is no theoretical support to show that the dynamical variabilities evaluated from such manipulated time series are still as trustworthy. Similar potential disruptions typically occur in other oscillatory physiological signals. We therefore expect that the combination of the proposed phenomenological model and the analysis method, SST, have great potential in dealing with broader physiological signals than the traditional approaches, especially when the scale of the signal is large and the determination of the landmarks is not easy.

In the introduction, we identified three drawbacks of presently used methods. We have already shown that the method proposed here does not suffer from the first two drawbacks: we can use much shorter time observation time observation windows, and we need not identify precisely the oscillating pattern to be detected. The third drawback we formulated was the relative poverty of the traditional landmark-to-landmark time interval series. The importance of keeping a richer description is illustrated by our method's use of variation in both frequency and amplitude in the ventilator weaning example.

Physiologically, the respiration is not only controlled by the neural respiratory center, but also controlled by the arterial chemoreceptors, lung vagal sensory receptors, lung mechanics, etc. In a normal subject, these control factors are integrated in a complex way which leads to the RRV [12] [14] [15]. Although we do not have a definite evidence, based on the reasoning about the relationship between the decreased HRV (or RRV) and the severity of disease [2] [4] [14]–[16], we hypothesize that the distinguishing power of WIN is a consequence of the possible disintegrity of the respiratory control factors, and this disintegrity leads to the decreased variability in the breathing pattern. This hypothesis indicates that the subject with decreased WIN is not completely ready for weaning, and explains why we observe the different patterns in the two groups. Also note that RRV is a different notion compared with the descriptive breathing parameters, like total volume, peak respiratory flow, total breath duration, and so on, which quantify the average behavior of one breath but not the complex integrity of the control factors.

This discussion would not be complete without listing the shortcomings of our approach. First, the phenomenological nature of our analysis limits the possibility to extract detailed underlying mechanisms leading to the variability and hence the prediction outcome. For example, we are not able (nor did we attempt) to distinguish if the observed variability is purely neural in origin, or if mechanical factors also play a role; finer modeling is needed to decide this, if possible. Second, from the viewpoint of estimating physiological dynamics, monitoring variability from the signal of one physiological subsystem, for example, the respiratory signal, is not sufficient: the physiological dynamics is the outcome of the complicated interactions between different physiological subsystems. Incorporating different simultaneously recorded biomedical signals, such as electrocardiograms, respiratory signal, blood pressure, and so on, should lead to a more informative description of the systematic dynamics. A study of how to extract the information on the interaction between different subsystems via the combination of the existing model and the SST algorithm is now ongoing. Specifically, although we now have a suitable method to extract information from different oscillatory physiological signals of different scales, finding their interactions and inferring more information remains an open problem. Third, the limitation inherent to the SST itself cannot be overlooked. Indeed, it is an intrinsic limitation of the SST that its estimation of the instantaneous frequency is less reliable when this instantaneous frequency has large local variations. To address this, we need a better theoretically rigorous approach to estimate the instantaneous frequency for such signals; work on this is ongoing. Finally, the study in the testbed has two limitations: it is retrospective, and the data have been collected for a small clinical population only, although the group of patients is homogeneous, in the sense that all patients are confirmed to be ready for weaning based on the RSBI. Yet it certainly warrants a follow-up prospective and large-scale clinical study to investigate its clinical applicability.

Despite these shortcomings, our example supports that the criterion we propose (in the form of the WIN) has the potential to assist physicians in assessing weaning readiness. The result encourages its application to other different kinds of oscillatory signals, in particular to those with (relatively) long periods. In conclusion, the proposed model and algorithm together efficiently relieve the difficulty shared by the traditional methods presently used to analyze physiological dynamics or more general oscillatory signals—the data length needed for analyzing the dynamics is significantly shortened, the effect of the inevitable noise is reduced, and the pattern of the oscillatory phenomenon does not play a significant role.

## Reallocation Technique and Synchrosqueezing Transform

The main mathematical tool we apply to analyze the oscillatory physiological signal is the newly developed, mathematically rigorous proved, and adaptive time-frequency (TF) analysis referred to as the synchrosqueezing transform (SST) [20] [21] [23] [27] [31] [32] [33] [34].

Besides the traditional analytic approach, for example, the Hilbert transform, there are many existing TF analysis method available to estimate $\phi^{\prime }(t)$ of the signal expressed in (9) [35]. Due to the Heisenberg uncertainty principal, the ambiguity in the TF representation is inevitable, and the reallocation technique was proposed for the sake of sharpening the TF representation [24]–[26] [34] [35] [36]. SST is a special reallocation technique. In the following, we start from giving the precise conditions and definitions about the functional class modeling the oscillatory physiological signal, and then discuss the reallocation technique and its special case SST. We mention that we can also consider STFT, but here we focus on the CWT to simplify the discussion.

Fix a Schwartz function $\psi$ so that $\hbox{supp }\widehat{\psi }\subset [1-\Delta,1+\Delta]$, where $0<\Delta <1$ which is commonly called the mother wavelet. Recall that the CWT [37] of a given $f(t)\in {\cal S}^{\prime }$ is defined by

$$W_f(a,b)=\int_{-\infty }^\infty f(t){1\over \sqrt{a}} \overline{\psi \left({t-b\over a} \right)} \hbox{d}t, \eqno{\hbox{(7)}}$$

where $a>0$ and $b\in {\BBR}$. Here, we follow the convention in the wavelet literature that $a$ means scale and $b$ means time. To ease the notation, the moments of $\psi$ are denoted as $I^{(k)}_i=\int_{\BBR}\vert x\vert^i\vert \psi^{(k)}(x)\vert \hbox{d}x$ for $k=0,1,\ldots$.

A continuous function is called an nonharmonic intrinsic mode function if it satisfies

$$f(t)=A(t)s(\phi (t)), \eqno{\hbox{(8)}}$$

where

I) $A\in C^1({\BBR})\cap L^\infty ({\BBR}), \phi {\kern-1pt}\in{\kern-1pt} C^2({\BBR}), \inf_{t\in {\BBR}} A(t){\kern-1pt}\ge{\kern-1pt} c_1{\kern-1pt}>0, \inf_{t\in {\BBR}}\phi^{\prime }(t){\kern-1pt}\ge{\kern-1pt} c_1>0, \sup_{t\in {\BBR}}A(t)<c_2, \sup_{t\in {\BBR}}\phi^{\prime }(t) < c_2, \vert A^{\prime }(t)\vert \le \epsilon \phi^{\prime }(t)$ and $\vert \phi^{\prime \prime }(t)\vert \le \epsilon \phi^{\prime }(t)$, for all $t$, and $\epsilon$ is a small parameter chosen in the model;
II) $s:[0,1]\rightarrow {\BBR}$ is $C^{1,\alpha }$, where $\alpha >1/2$, and $\hbox{1}$-periodic function with unit $L^2$ norm, $\vert \widehat{s}(k)\vert \le \delta \vert \widehat{s}(1)\vert$ for all $k\ne 1$, where $\delta \ge 0$ is a small parameter, and $\sum_{n>D}\vert n\widehat{s}(n)\vert \le \theta$ for some small parameter $\theta \ge 0$ and $D\in {\BBN}$.

Note that we assume that the signal has just one component, unlike the case in previous studies wherein the signals were considered to include several components [22]. We shall call $\phi$ the phase function of the signal $f(t)$ and the derivative $\phi^{\prime }(t)$ of the phase function the instantaneous frequency (IF) of $f(t)$. Next, we model the measured physiological signal as

$$Y(t)=A(t)s(\phi (t))+T(t)+\sigma (t)\Phi (t), \eqno{\hbox{(9)}}$$

where $s(t), A(t)$, and $\phi (t)$ satisfy (I) and (II) and the following condition is satisfied:

III) $T:{\BBR}\rightarrow {\BBR}$ is in $C^1({\BBR})$ so that its Fourier transform exists in the distribution sense, and $\vert T(\psi_{a,b})\vert,\,\vert T^{\prime }(\psi_{a,b})\vert \le C_T\epsilon$ for all $b\in {\BBR}$ and $a\in (0,{1+\Delta \over c_1}]$, for some $C_T\ge 0$ [21].
IV) $\Phi (t)$ is a stationary generalized random process (GRP) or “almost” stationary GRP [21] independent of $A(t)s(\phi (t))$, which is introduced to model the measurement error. To be more specific, the power spectrum $\hbox{d}\eta$ of the given GRP $\Phi$ satisfies $\int (1+\vert \xi \vert)^{-2l}\hbox{d}\eta <\infty$ for some $l>0$. Also assume $\sigma \in C^\infty$ so that $\Vert \sigma \Vert_{L^\infty }\ll 1$ and $\epsilon_\sigma :={\rm max}_{\ell =1,\ldots,{\rm max}\{1,l\} }\{\Vert \sigma^{(\ell)}\Vert_{L^\infty }\} \ll 1$, and $\hbox{var}\Phi (\psi)=1$.

Now we discuss the reallocation technique and the SST. Take the CWT of a given observation $Y$ in (9)

$$W_Y(a,b)=\int_{-\infty }^\infty Y(t){1\over \sqrt{a}} \overline{\psi \left({t-b\over a} \right)}\hbox{d}t. \eqno{\hbox{(10)}}$$

Note that (10) is well defined since $\sigma (t)\Phi (t)$ is GRP and by the assumption of $T$. The reallocation technique “sharpens” $W_Y$ by “reallocating” the value at $(t,\xi)$ to a different point $(t^{\prime },\xi^{\prime })$ according to some reassignment rules [25] [26] [34] [35], where $t^{\prime }$ might be different from $t$. In contrast to the reallocation technique, in the SST, $W_Y$ is reallocated from $(t,\xi)$ to a different point $(t,\xi^{\prime })$ according to a different reassignment rules which only reallocates the frequency axis and preserves the time information. Note that it is important in biomedical application, in particular when prediction is the purpose, since in general we do not have the future information.

Here we detail the SST, which is separated into three steps. First, calculate $W_Y(a,b)$. Second, calculate the reallocation rule $\omega_Y$ defined on ${\BBR}^+\times {\BBR}$:

$$\omega_Y(a,b):=\!\left\{\!\matrix{\displaystyle {-i\partial_bW_Y(a,b)\over 2\pi W_Y(a,b)}\hfill & \hbox{when}\; \vert W_Y(a,b)\vert \ne 0; \hfill\cr -\infty\hfill &\hbox{when}\; \vert W_Y(a,b)\vert =0.\hfill}\right. \eqno{\hbox{(11)}}$$

Third, the SST of $Y(t)$ is defined by reallocating the coefficients of $W_Y(a,b)$ according to the reallocation rule $\omega_f(a,b)$:

$$\eqalignno{S^{\Gamma }_Y(b,\xi) &:= \lim_{\alpha >0,\alpha \rightarrow 0}\int _{\{(a,b): \vert W_Y(a,b)\vert \ge {\Gamma }\} }{1\over \alpha } h\!\left({\vert \omega_Y(a,b)-\xi \vert \over \alpha } \right)\cr &\quad\times W_Y(a,b)a^{-3/2}\hbox{d}a &{\hbox{(12)}} }$$

where $(b,\xi)\in {\BBR}\times {\BBR}^+, \Gamma >0$ is the threshold chosen by the user, $h$ is a kernel function which is smooth enough and decays fast enough. Intuitively, at each time point $t$, we collect all CWT coefficients with scales $a$ at which $\omega_Y(a,b)$ is close to $\xi$ and put them in the $(b,\xi)$ slot. As is shown in [20]–[22], $S^{\Gamma }_Y(b,\xi)$ will only have dominant values around $\phi^{\prime }(b)$. This property allows us an accurate estimate of $\phi^{\prime }$ by, for example, the curve extraction technique. The estimated $\phi^{\prime }$ is denoted by $\widetilde{\phi }^{\prime }$.

With the estimated $\phi^{\prime }$, we can estimate $A(t)$ and $\phi (t)$ in (9) by the following reconstruction formula. Define

$$\widetilde{R}(t):={\cal R}_\psi^{-1}{\mathfrak {Re}}\int_{\{\xi : \vert \phi^{\prime }(t)-\xi \vert \le \epsilon^{1/3}\} }S_Y^\Gamma (t,\xi)\hbox{d}\xi, \eqno{\hbox{(13)}}$$

where ${\cal R}_\psi :=\int {\widehat{\psi }(\zeta)\over \zeta } \hbox{d}\zeta$ and ${\mathfrak {Re}}$ means taking the real part. Based on the theorem in [20]–[22], the estimator of $A(t)$ is defined as $\widetilde{A}(t):=\vert \widetilde{R}(t)\vert$. Then an estimator for $\phi (t)$, denoted as $\widetilde{\phi }(t)$, can be obtained by unwrapping the phase of the complex-valued signal $\widetilde{R}(t)/\widetilde{A}(t)$. As is shown in [21, Th. 3.1], these estimators are accurate and are robust to the existence of the trend $T(t)$ and noise $\sigma (t)\Phi (t)$.

Notice that we can interpret (13) as an adaptive band-pass filter. Indeed, at each time $t$, the estimation of $\phi^{\prime }(t)$ by SST can be interpreted as the main frequency region corresponding to the physiological signal at time $t$. Then, the reconstruction of the physiological signal at time $t$ is based on the chosen frequency band with bandwidth $\epsilon^{1/3}$.

We summarize the theoretical results of SST relevant to this paper, and refer the reader to [20] [21] [23] for the precise statement of the theorem.

P1: SST is robust to the several different kinds of noise, which might be slightly nonstationary. Thus, we are able to accurately estimate the IF and AM [21] [23].
P2: Since SST is local in nature, we are able to detect components that do not exist all the time and hence the dynamical behavior of the signal [20] [21].
P3: The time-frequency representation $S^{\Gamma }_Y(b,\xi)$ is visually informative. See Fig. 3 for example.
P4: SST is “adaptive” to the data in the sense that the error in the estimation depends only on the first three moments of the mother wavelet instead of the profile of the mother wavelet. See [23, Fig. 6] for a visual demonstration.
P5: The existence of the trend modeled in (9) do not interfere with the SST. Thus, we are able to separate the trend and periodic components. Note that a smooth function $T\in C^\infty \cap {\cal S}^{\prime }$ so that its Fourier transform $\widehat{T}$ is compactly supported in a small interval around $\hbox{0}$ is a special case.

## Numerical Implementation of SST

In this section, we provide the numerical implementation detail of SST. The MATLAB code is available in http://sites.google.com/site/hautiengwu/ and we refer the readers to [23] for more implementation details.

Take a discretization of (9), denoted as ${\bm Y}\in {\BBR}^N$, with the sampling period $\tau >0$ from time $\tau$ to time $N\tau$, that is, ${\bm Y}(n)=Y(n\tau)$. Here, we have to be careful about the meaning of $Y(n\tau)$ when $\Phi$ is a GRP. Theoretically, when $\Phi$ is in the general sense, for example, the differentiation of the standard Brownian motion, it does not make sense to directly evaluate $\Phi$ at a particular time [38]. Also, as is discussed in [21] and the references therein, the relationship between the continuous random process and discrete time series is not always one to one. For example, not every autoregressive and moving average time series (ARMA) can be embedded into a continuous ARMA random process. Thus, in the discrete case, sometimes the random error term needs to be modeled separately, that is, ${\bm Y}\in {\BBR}^N$ satisfies

$${\bm Y}(n)=f(n\tau)+T(n\tau)+\sigma (n\tau)\Psi (n),$$

where $n=1,\ldots,N, f, T$ and $\sigma$ satisfy conditions (I), (II) and (III), and $\Psi$ is a zero-mean stationary time series, which can be taken as an ARAM time series, generalized autoregressive conditional heteroskedasticity time series, etc. In the following, to simplify the discussion, we assume that the discretization of (9) can be carried out directly, and refer the reader to [21] for more theoretical results about discretization.

In practice, to prevent boundary effects, we pad ${\bm Y}$ on both sides by reflecting the signal near the boundary so that its length is $N^{\prime }=2^{L+1}$, where $L$ is the minimal integer such that $N^{\prime }>N$. Although it works well in practice, we emphasize that doing so is not the optimal solution. To ease the notation, in the following, we use the same notation ${\bm Y}$ to denote the padded signal and $N$ to indicate the length of the padded signal.

### VI Step 1: Numerically Implement the CWT

For the sake of implementing (10), we take the scales $a_j=2^{j/n_v}\tau, j=1,\ldots, Ln_v$, where $n_v$ is the “voice number” chosen by the user. In practice, $n_v=\hbox{32}$ performs well. We denote the implemented CWT as a $N\times n_a$ matrix ${\bm W}_{\bm Y}$. We may directly follow the code available from wavelab ^2^ http://www-stat.stanford.edu/∼wavelab/ which implements CWT in the Fourier domain, or directly implement it by convolution in the time domain.

### A. Step 2: Numerically Implement $\omega_Y(a,b)$

To numerically implement the reallocation rule $\omega_Y(a,b)$ (11), we have to discretize $\partial_b W_Y(a,b)$. It is evaluated directly by finite difference at the time axis $b$ and we denote the result as a $N\times n_a$ matrix ${\bm \partial}_{\bm b}{\bm W}_{\bm Y}$. The $\omega_Y(a,b)$ is then implemented as a $N\times n_a$ matrix ${\bm \omega}_{\bm Y}$ by the following entrywise calculation:

$${\bm \omega}_{\bm Y}(i,j)=\left\{\matrix{\displaystyle {-i{\bm \partial}_{\bm b}{\bm W}_{\bm Y}(i,j)\over 2\pi {\bm W}_{\bm Y}(i,j)}\hfill &\hbox{when }{\bm W}_{\bm Y}(i,j)\ne 0 \hfill\cr -\infty\hfill &\hbox{when }{\bm W}_{\bm Y}(i,j)=0.\hfill}\right.$$

Note that although ${\bm W}_{\bm Y}(i,j)=0$ is numerically unstable, but this unstability will be taken care in the next step.

### B. Step 3: Numerically Implement $S^\Gamma_Y(b,\xi)$

To implement the Synchrosqueezing transform $S^\Gamma_Y(b,\xi)$ (12), we discretize the frequency domain $[{1\over N\tau },{1\over 2\tau }]$ by equally spaced intervals of length $\Delta_{\xi }={1\over N\tau }$. Here, ${1\over N\tau }$ and ${1\over 2\tau }$ are the minimal and maximal frequencies detectable by the Fourier transform theorem. Note that the dc (direct current) term is not considered in SST. Denote $n_{\xi }=\lfloor {{1\over 2\tau } -{1\over N\tau } \over \Delta_{\xi}} \rfloor$, which is the number of the discretization of the frequency axis. Fix $\Gamma >0$, the discretized $S^\Gamma_Y(b,\xi)$, denoted by a $N\times n_{\xi }$ matrix ${\bm S}_{\bm Y}$, is evaluated by

$${\bm S}_{\bm Y}(i,j)=\sum_{{{{{k: \vert {\bm \omega}_{\bm Y}(i,k)-j\Delta_{\xi }\vert \le \Delta_{\xi }/2,\atop \vert {\bm W}_{\bm Y}(i,j)\vert \ge \Gamma}}}}} {\log (2) \sqrt{a_j}\over \Delta_{\xi } n_v} {\bm W}_{{\bm Y}}(i,k),$$

where $i=1,\ldots,N$ and $j=1,\ldots,n_\xi$. Notice that the number $\Gamma$ is a hard thresholding parameter, which is chosen to reduce the influence of noise and numerical error encountered when ${\bm W}_{\bm Y}(i,j)$ is small. We choose $\Gamma =1e-3$ in this study. If the error is Gaussian white noise, the choice of $\Gamma$ is suggested in [23]. In general, more study is needed to adaptively choose $\Gamma$ from a given time series.

To visually see the time frequency representation of the SST, we may directly plot the $N\times n_{\xi }$ matrix ${\bm R}_{\bm Y}$ defined by

$${\bm R}_{\bm Y}(i,j):=\vert {\bm S}_{\bm Y}(i,j)\vert^2$$

for all $i=1,\ldots,N$ and $j=1,\ldots,n_\xi$, and observe its behavior.

### C. Step 4: Estimate IF, AM, and Trend From ${\bm S}_{\bm Y}$

According to the theoretical statement, the SST time frequency representation will be dominant in the IF [21] [23]. Thus, we estimate IF by fitting a discretized curve ${\bm c}^*\in Z_{n_{\xi}}^{N}$, where $Z_{n_{\xi}}=\{1,\ldots,n_{\xi }\}$ indexes the discretized frequency axis, to the dominant area of ${\bm S}_{\bm Y}$. Precisely, we maximize the following functional over ${\bm c}\in Z_{n_{\xi}}^{N}$:

$$\eqalignno{{\bm c}^*&:=\mathop{\hbox{argmax}}\limits_{{\bm c}\in Z_{n_{\xi}}^{N}}\sum_{m=1}^{N} \log \left({\vert {\bm S}_{\bm Y}(m,{\bm c}(m))\vert \over \sum_{i=1}^{n_{\xi}}\sum_{j=1}^{N}\vert {\bm S}_{\bm Y}(j,i)\vert } \right) \cr &\quad-\lambda \sum_{m=2}^{N} \vert {\bm c}_m-{\bm c}_{m-1}\vert^2, &\hbox{(14)}}$$

where $\lambda >0$ determines the regularity of the estimated curve. The first term is aimed to capture the maximal value of ${\bm S}_{\bm Y}$ at each time and the second term is aimed to impose regularity of the extracted curve. In this study, we simply choose $\lambda =1$. Denote the maximizer of the functional in (14) as ${\bm c}^*\in {\BBR}^N$. Then SSTIF, denoted as ${\bm \phi^{\prime}}\in {\BBR}^N$, is given by

$${\bm \phi^{\prime}}(n):={\bm c}^*(n)\Delta_{\xi },$$

where $n=1,\ldots,N$. Here, ${\bm \phi^{\prime}}(n)$ is the estimated IF at time $n\tau$. With ${\bm c}^*$, SSTAM, denoted as ${\bm A}\in {\BBR}^N$, is evaluated by

$$ \eqalignno{ &{\bm f}_{\bf 1}(n):= { 2\over {\cal R}_{\psi }} \Delta_\xi \sum_{i={\bm c}^*(n)-\lfloor \Delta /\Delta_\xi \rfloor }^{{\bm c}^*(n)+\lfloor \Delta /\Delta_\xi \rfloor } {\bm S}_{\bm Y}(n,i), \cr &{\bm A}(n):=\vert {\bm f}_1(n)\vert, }
$$

where $n=1,\ldots,N, {\bm f}_{\bf 1}(n)\in {\BBC}$, and $\Re$ is the real part. The phase function $\phi$ is then estimated by unwrapping ${\bm f}_{\bf 1}(n)/\vert {\bm f}_{\bf 1}(n)\vert$. Note that estimating the phase by integrating the estimated IF is not recommended since the error might be accumulated. Lastly, the trend $T(t)$ at time $t=n\tau$ can be estimated by

$${\bm T}(n):={\bm Y}_n-\Re {2\over {\cal R}_{\psi}} \Delta_\xi \sum_{i=\lfloor \xi_{{\rm l}}/\Delta \xi \rfloor }^{n_\xi } {\bm S}_{\bm Y}(n,i),$$

where ${\bm T}\in {\BBR}^N$ and $\xi_{{\rm l}}$ is chosen by the user, if recovering the trend is necessary.

## References

[1] Bakris G., “Hypertension in 2011: New insights—From risk factors to treatment implications,” Nat. Rev. Cardiol., vol. 9, pp. 75–77, 2011. 22158080 10.1038/nrcardio.2011.202

[2] Ferrer R. and Artigas A., “Physiologic parameters as biomarkers: What can we learn from physiologic variables and variation?,” Crit. Care. Clin., vol. 27, pp. 229–240, 2011. 21440198 10.1016/j.ccc.2010.12.008

[3] Benchetrit G., “Breathing pattern in humans: Diversity and individuality,” Respir. Physiol., vol. 122, no. 2–3, pp. 123–129, 2000. 10967339 10.1016/s0034-5687(00)00154-7

[4] Vanderlei L., Pastre C., Hoshi R., Carvalho T., and Godoy M., “Basic notions of heart rate variability and its clinical applicability,” Rev. Bras. Cir. Cardiovasc., vol. 24, pp. 205–217, 2009. 19768301 10.1590/s0102-76382009000200018

[5] Takeda N. and Maemura K., “Circadian clock and cardiovascular disease,” J. Cardiol., vol. 57, pp. 249–256, 2011. 21441015 10.1016/j.jjcc.2011.02.006

[6] Wang X., “Neurophysiological and computational principles of cortical rhythms in cognition,” Physiol. Rev., vol. 90, pp. 1195–1268, 2010. 20664082 10.1152/physrev.00035.2008 PMC2923921

[7] Golombek D. and Rosenstein R., “Physiology of circadian entrainment,” Physiol. Rev., vol. 90, pp. 1063–1102, 2010. 20664079 10.1152/physrev.00009.2009

[8] Pigolotti S., Krishna S., and Jensen M., “Oscillation patterns in negative feedback loops,” Proc. Nat. Acad. Sci. USA, vol. 104, pp. 6533–6537, 2007. 17412833 10.1073/pnas.0610759104 PMC1871820

[9] Ahmad S., Tejuja A., Newman K., R Z., and Seely A., “Clinical review: A review and analysis of heart rate variability and the diagnosis and prognosis of infection,” Crit. Care, vol. 13, 2009. 10.1186/cc8132 PMC2811891 20017889

[10] Casaseca-de-la Higuera P., Martín-Fernández M., and Alberola-López C., “Weaning from mechanical ventilation: a retrospective analysis leading to a multimodal perspective,” IEEE Trans. Biomed. Eng., vol. 53, no. 7, pp. 1330–1345, Jul. 2006. 16830937 10.1109/TBME.2006.873695

[11] Brack T., Jubran A., and Tobin M., “Dyspnea and decreased variability of breathing in patients with restrictive lung disease,” Amer. J. Respir. Crit. Care. Med., vol. 165, pp. 1260–1264, 2002. 11991875 10.1164/rccm.2201018

[12] Engoren M., “Approximate entropy of respiratory rate and tidal volume during weaning from mechanical ventilation,” Crit. Care Med., vol. 26, pp. 1817–1823, 1998. 9824073 10.1097/00003246-199811000-00021

[13] Bien M.-Y., Yien H.-W., Hseu S.-S., Wang J.-H., and Kou Y.-R., “Instability of spontaneous breathing patterns in patients with persistent vegetative state,” Respir. Physiol. Neurobiol., vol. 145, no. 2–3, pp. 163–175, 2005. 15705532 10.1016/j.resp.2004.09.007

[14] Bien M.-Y., Hseu S.-S., Yien H.-W., Kuo I.-T., Lin Y.-T., Wang J.-H., and Kou Y. R., “Breathing pattern variability: A weaning predictor in postoperative patients recovering from systemic inflammatory response syndrome,” Intens. Care Med., vol. 30, pp. 241–247, 2004. 10.1007/s00134-003-2073-8 14647889

[15] Bien M.-Y., Lin Y.-S., Shih C.-H., Yang Y.-L., Lin H.-W., Bai K.-J., Wang J.-H., and Kou Y.R., “Comparisons of predictive performance of breathing pattern variability measured during t-piece, automatic tube compensation, and pressure support ventilation for weaning intensive care unit patients from mechanical ventilation,” Crit. Care Med., vol. 39, pp. 2253–2262, 2011. 21666447 10.1097/CCM.0b013e31822279ed

[16] Wysocki M., Cracco C., Teixeira A., Mercat A., Diehl J., Lefort Y., Derenne J., and Similowski T., “Reduced breathing variability as a predictor of unsuccessful patient separation from mechanical ventilation,” Crit. Care Med., vol. 34, pp. 2076–2083, 2006. 16755257 10.1097/01.CCM.0000227175.83575.E9

[17] Malik M. and Camm A. J., Heart Rate Variability, New York, NY USA: Wiley, 1995.

[18] Engoren M., Courtney S., and Habib R., “Effect of weight and age on respiratory complexity in premature neonates,” Comput. Cardiol., vol. 106, pp. 766–773, 2009. 10.1152/japplphysiol.90575.2008 19036892

[19] Moody G., Mark R., Zoccola A., and Mantero S., “Derivation of respiratory signals from multi-lead ecgs,” Comput. Cardiol., vol. 12, pp. 113–116, 1985.

[20] Daubechies I., Lu J., and Wu H.-T., “Synchrosqueezed wavelet transforms: An empirical mode decomposition-like tool,” Appl. Comput. Harmon. Anal., vol. 30, no. 2, pp. 243–261, 2011.

[21] Chen Y.-C., Cheng M.-Y., and Wu H.-T., “Nonparametric and adaptive modeling of dynamic seasonality and trend with heteroscedastic and dependent errors,” J. Roy. Stat. Soc. B, 2013, to be published.

[22] Wu H.-T., “Instantaneous frequency and wave shape functions (I),” Appl. Comput. Harmon. Anal., vol. 35, pp. 181–199, 2013.

[23] Thakur G., Brevdo E., Fuckar N. S., and Wu H.-T., “The synchrosqueezing algorithm for time-varying spectral analysis: Robustness properties and new paleoclimate applications,” Signal Process., vol. 93, pp. 1079–1094, 2013.

[24] Kodera K., Gendrin R., and Villedary C., “Analysis of time-varying signals with small bt values,” IEEE Trans. Acoust., Speech, Signal Process., vol. 26, no. 1, pp. 64–76, Feb. 1978.

[25] Auger F. and Flandrin P., “Improving the readability of time-frequency and time-scale representations by the reassignment method,” IEEE Trans. Signal Process., vol. 43, no. 5, pp. 1068–1089, 5 1995.

[26] Chassande-Mottin E., Auger F., and Flandrin P. Time-frequency/time-scale reassignment Wavelets and Signal Processing (ser. Applied Numerical Harmonic Analysis), Boston, MA USA: Birkhäuser, 2003, pp. 233–267.

[27] Daubechies I. and Maes S. A nonlinear squeezing of the continuous wavelet transform based on auditory nerve models Wavelets in Medicine and Biology Aldroubi A., and Unser M., Eds., Boca Raton, FL USA: CRC Press, 1996, pp. 527–546.

[28] Pingleton S. Complications associated with mechanical ventilation Principles and Practice of Mechanical Ventilation Tobin M. J., Ed., New York, NY USA: McGraw-Hill, 1994, pp. 775–792.

[29] Cook D., Meade M., and Perry A., “Qualitative studies on the patient's experience of weaning from mechanical ventilation,” Chest, vol. 120, pp. 469s–473s, 2001. 11742967 10.1378/chest.120.6_suppl.469s

[30] Yang K. and Tobin M., “A prospective study of indexes predicting the outcome of trials of weaning from mechanical ventilation,” N. Engl. J. Med., vol. 324, no. 21, pp. 1445–1450, 1991. 2023603 10.1056/NEJM199105233242101

[31] Wu H.-T., Flandrin P., and Daubechies I., “One or two frequencies? The synchrosqueezing answers,” Adv. Adapt. Data Anal., vol. 3, pp. 29–39, 2011.

[32] Thakur G. and Wu. H.-T., “Synchrosqueezing-based recovery of instantaneous frequency from nonuniform samples,” SIAM J. Math. Anal., vol. 43, pp. 2078–2095, 2010.

[33] Wu H.-T., Chan Y.-H., Lin Y.-T., and Yeh Y.-H., “Using synchrosqueezing transform to discover breathing dynamics from ECG signals,” Appl. Comput. Harmon. Anal., 2013, to be published.

[34] Auger F., Flandrin P., Lin Y.-T., McLaughlin S., Meignen S., Oberlin T., and Wu H.-T., “Recent advances in time-frequency reassignment and synchrosqueezing,” IEEE Trans. Signal Process., 2013, to be published.

[35] Flandrin P., Time-Frequency/Time-Scale Analysis, New York, NY USA: Academic, 1999, vol. 10.

[36] Chassande-Mottin E., Daubechies I., Auger F., and Flandrin P., “Differential reassignment,” IEEE Signal Process. Lett., vol. 4, no. 10, pp. 293–294, Oct. 1997.

[37] Daubechies I., Ten Lectures on Wavelets, Philadelphia, PA USA: Society for Industrial and Applied Mathematics, 1992.

[38] Gel'fand I. and Vilenkin N. Y., Generalized Function Theory, New York, NY USA: Academic, 1964, vol. 4.

